# Supplementary material for: Phylogenetic diversity and North Andean block conservation
Source: PeerJ. 2023 Dec 6;11:e16565. doi: 10.7717/peerj.16565 (PMC10710123; doi:10.7717/peerj.16565)
Supplement: Supplemental Information 1 — All the species included in the study, with each species showing its corresponding branch length and indicating whether it is endemic or widespread. [file peerj-11-16565-s001.pdf]

Species	Branch Length	Distribution
Acalypha_platyphylla	0.01058233	Widespread
Acalypha_diversifolia	3.90000000002888e-07	Widespread
Acalypha_macrostachya	0.006697	Widespread
Acalypha_cuspidata	0.00991001	Widespread
Acalypha_wilkesiana	5.1919999999997e-05	Widespread
Acalypha_hispida	0.00220306	Widespread
Acalypha_schiedeana	0.00284686	Widespread
Acalypha_alopecuroidea	0.00243688	Widespread
Acalypha_ostryifolia	1.00000000002876e-07	Endemic
Acalypha_setosa	2.90000000000012e-07	Widespread
Anolis_huilae	0.145122400000002	Endemic
Anolis_fitchi	0.151220500000001	Widespread
Anolis_calimae	0.173309930000002	Endemic
Anolis_frenatus	1.30000000098107e-06	Widespread
Anolis_latifrons	0.0186032199999993	Widespread
Anolis_princeps	0.0239916200000003	Widespread
Anolis_maculigula	0.09100866	Widespread
Anolis_danieli	0.100651859999999	Widespread
Anolis_chocorum	0.0855914800000015	Widespread
Anolis_fraseri	0.0996838500000017	Widespread
Anolis_peraccae	0.1399215	Widespread
Anolis_chloris	1.30000000098107e-06	Widespread
Anolis_ventrimaculatus	0.119918650000002	Widespread
Anolis_aequatorialis	0.0688228600000009	Widespread
Anolis_anoriensis	0.0746898199999997	Endemic
Anolis_gemmosus	0.0679977799999989	Endemic
Anolis_jacare	0.0428015299999984	Widespread
Anolis_anatoloros	0.027492849999998	Endemic
Anolis_punctatus	0.127433710000002	Widespread
Anolis_transversalis	0.122802670000002	Widespread
Anolis_heterodermus	0.022462240000003	Widespread
Anolis_inderenae	0.0465170600000029	Endemic
Anolis_vanzolinii	0.0193516100000011	Endemic
Anolis_ortonii	0.162928430000001	Widespread
Anolis_tropidolepis	0.0720300400000013	Endemic
Anolis_humilis	0.0583418999999985	Endemic
Anolis_poecilopus	0.0741838199999982	Widespread
Anolis_tropidogaster	0.0736687099999997	Widespread
Anolis_trachyderma	0.0871046	Widespread
Anolis_fuscoauratus	0.0388802200000029	Widespread
Anolis_chrysolepis	0.158657120000001	Widespread
Anolis_bitectus	0.104082600000002	Widespread
Anolis_gracilipes	0.13678359	Widespread

Anolis_biporcatus	0.14866726	Widespread
Anolis_scypheus	0.10147456	Widespread
Anolis_bombiceps	0.0582345399999973	Endemic
Anolis_auratus	0.186090570000001	Widespread
Aotus_lemurinus	0.00242505000000001	Widespread
Aotus_griseimembra	0.00856040999999999	Endemic
Aotus_vociferans	0.0200485	Widespread
Aotus_trivirgatus	0.0183721	Widespread
Ara_ararauna	0.00405641	Widespread
Ara_severus	0.0049336	Widespread
Ara_militaris	0.00104014	Widespread
Ara_ambiguus	0.00071864	Widespread
Ara_chloropterus	0.00263039	Widespread
Ara_macao	0.00180194	Widespread
Amazona_festiva	0.00294719	Widespread
Amazona_farinosa	0.00346528	Widespread
Amazona_barbadensis	0.00130522	Widespread
Amazona_ochrocephala	0.000534659999999999	Widespread
Amazona_autumnalis	0.00247624	Widespread
Amazona_amazonica	0.000605410000000001	Widespread
Centurio_senex	0.05442605	Widespread
Sphaeronycteris_toxophyllum	0.03757063	Widespread
Ametrida_centurio	0.0266516	Widespread
Enchisthenes_hartii	0.20945486	Widespread
Artibeus_concolor	0.06945749	Endemic
Artibeus_lituratus	0.00722350999999999	Widespread
Artibeus_obscurus	0.04774508	Widespread
Artibeus_planirostris	0.02480912	Widespread
Artibeus_jamaicensis	0.03997027	Widespread
Artibeus_fraterculus	0.04031462	Widespread
Dermanura_glauca	0.06718117	Widespread
Dermanura_bogotensis	0.04112457	Widespread
Dermanura_cinerea	0.07458572	Widespread
Dermanura_rosenbergi	0.06416862	Endemic
Dermanura_watsoni	0.05376144	Widespread
Dermanura_phaeotis	0.09830289	Widespread
Dermanura_tolteca	0.04004407	Widespread
Dermanura_rava	0.04589421	Widespread
Dermanura_anderseni	0.04574688	Widespread
Alouatta_seniculus	0.00379845000000001	Widespread
Alouatta_palliata	0.01066338	Widespread
Ateles_belzebuth	0.01608155	Widespread
Ateles_hybridus	0.02153232	Widespread
Ateles_geoffroyi	0.02069816	Widespread

Ateles_fusciceps	0.01146733	Widespread
Lagothrix_lugens	0.00152331	Widespread
Lagothrix_poeppigii	0.00545224999999999	Endemic
Atractus_wagleri	0.01893164	Endemic
Atractus_schach	0.02548019	Endemic
Atractus_duboisi	0.0176333	Endemic
Atractus_resplendens	0.01549281	Endemic
Atractus_elaps	0.03058121	Widespread
Atractus_major	0.04241057	Widespread
Atractus_dunni	0.00848931	Endemic
Atractus_iridescens	0.00565176000000001	Widespread
Atractus_badius	0.04406756	Endemic
Atractus_flammigerus	0.02575977	Widespread
Bactris_schultesii	0.0120944	Endemic
Bactris_coloradonis	0.00391791	Widespread
Bactris_concinna	0.00402285999999999	Endemic
Bactris_coloniata	2.99199999999888e-05	Widespread
Bactris_guineensis	2.14400000000114e-05	Widespread
Bactris_brongniartii	0	Widespread
Bactris_major	7.88999999999929e-05	Widespread
Bactris_simplicifrons	0.08718455	Widespread
Bactris_setulosa	0.09885101	Widespread
Bactris_maraja	0.03471586	Widespread
Bactris_gasipaes	0.01599592	Widespread
Bactris_barronis	1.40000000009577e-07	Widespread
Marchantia_chenopoda	0.00366623999999999	Widespread
Marchantia_polymorpha	0.00929175999999998	Widespread
Marchantia_paleacea	0.00630018999999998	Widespread
Monoclea_forsteri	0.00676584000000002	Widespread
Monoclea_gottschei	0.01339617	Widespread
Bazzania_affinis	0.02361725	Widespread
Bazzania_tricrenata	0.00123364999999998	Endemic
Bomarea_hieronymi	0.0253711	Widespread
Bomarea_linifolia	0.0103295299999999	Widespread
Bomarea_edulis	0.00165141000000002	Widespread
Bomarea_densiflora	1.99999999894729e-08	Widespread
Bomarea_nervosa	8.25000000004295e-06	Endemic
Bomarea_ovata	0.00354958999999999	Widespread
Bomarea_purpurea	1.4019999999948e-05	Widespread
Bomarea_glaucescens	4.81500000000246e-05	Widespread
Bomarea_dissitifolia	8.4000000000195e-07	Endemic
Bomarea_perglabra	6.99999999076439e-08	Endemic
Bomarea_spissiflora	0.00296056	Endemic
Bomarea_angulata	2.1009999999988e-05	Widespread

Bomarea_uncifolia	0.03315957	Widespread
Bomarea_crassifolia	0.00371723000000002	Widespread
Bomarea_multiflora	0.01055055	Widespread
Bomarea_hirsuta	0.01530354	Widespread
Bomarea_diffracta	0.00349382999999992	Widespread
Bomarea_patinii	0.00801657999999994	Widespread
Bomarea_bredemeyerana	0.0116238200000001	Widespread
Bomarea_acutifolia	4.04999999992217e-06	Widespread
Bomarea_pardina	0.00354840000000001	Widespread
Bomarea_patacocensis	0.00876496000000004	Widespread
Bomarea_salsilla	0.01662992	Endemic
Bomarea_setacea	0.00698544000000001	Widespread
Bomarea_pauciflora	0.0936462	Widespread
Bomarea_caucana	0.00495634	Endemic
Bombus_excellens	0.01505659	Widespread
Bombus_pullatus	0.00355457999999997	Widespread
Bombus_transversalis	0.00365771999999998	Widespread
Bombus_rubicundus	0.01748119	Widespread
Bombus_funebris	0.01570856	Widespread
Bombus_melaleucus	0.00811919999999999	Widespread
Bombus_robustus	0.00564949999999997	Widespread
Bombus_hortulanus	0.00135552	Widespread
Caesalpinia_coriaria	0.01331073	Widespread
Caesalpinia_pluviosa	0.00523631000000002	Widespread
Caesalpinia_cassioides	0.00262693000000003	Widespread
Caesalpinia_pulcherrima	0.0146950699999999	Widespread
Caesalpinia_spinosa	0.00844794000000004	Widespread
Caesalpinia_crista	0.00750745999999991	Widespread
Caesalpinia_bonduc	0.00521606000000008	Widespread
Calamagrostis_rupestris	0.00167879999999998	Endemic
Calamagrostis_rigida	9.00000000081391e-08	Widespread
Calamagrostis_tarmensis	6.39999999996199e-07	Widespread
Calceolaria_ericoides	0.00647003000000002	Widespread
Calceolaria_comosa	0.00173909000000005	Endemic
Calceolaria_sericea	0.00143285999999998	Widespread
Calceolaria_perfoliata	0.00427993000000004	Widespread
Calceolaria_lanata	0.00173967000000008	Widespread
Calceolaria_pavonii	0.000872059999999841	Endemic
Calceolaria_tripartita	0.00837733000000007	Widespread
Calceolaria_chelidonioides	0.00174045999999994	Widespread
Calceolaria_dichotoma	0.0117430299999999	Endemic
Calceolaria_pedunculata	0.01013917	Widespread
Calceolaria_lehmanniana	0.00723406999999998	Widespread
Calceolaria_helianthemoides	0.00143685999999987	Endemic

Calceolaria_lavandulifolia	0.00423597000000009	Endemic
Calceolaria_purpurascens	0.000881560000000059	Widespread
Calceolaria_dilatata	1.30000000009289e-06	Widespread
Calceolaria_brachiata	0.01734459	Widespread
Calceolaria_nivalis	5.07000000005142e-06	Widespread
Calceolaria_fusca	0.00328011999999989	Endemic
Calceolaria_microbefaria	0.00143249000000001	Widespread
Calceolaria_phaeotricha	0.00377991999999994	Endemic
Calceolaria_rosmarinifolia	0.00526526000000005	Widespread
Calceolaria_gossypina	1.30000000009289e-06	Widespread
Calceolaria_martinezii	0.00262958000000002	Widespread
Calceolaria_spruceana	0.00349796999999996	Widespread
Calceolaria_lamiifolia	1.29999999987085e-06	Widespread
Calceolaria_penlandii	1.29999999987085e-06	Widespread
Caligo_idomeneus	0.02480297	Widespread
Caligo_atreus	0.01442915	Widespread
Caligo_eurilochus	0.00977135999999999	Widespread
Caligo_illioneus	0.01986481	Widespread
Caligo_teucer	0.09691979	Widespread
Caligo_oedipus	0.01156411	Widespread
Caligo_brasiliensis	0.00136174	Widespread
Caligo_telamonius	0.00519059999999999	Endemic
Speothos_venaticus	0.0603245	Widespread
Atelocynus_microtis	0.0125960000000001	Endemic
Lycalopex_culpaeus	0.00564083000000004	Endemic
Lycalopex_griseus	0.00395999999999996	Endemic
Cerdocyon_thous	0.04602633	Widespread
Urocyon_cinereoargenteus	0.07764924	Widespread
Canthon_subhyalinus	0.01216895	Widespread
Canthon_luteicollis	0.01608696	Widespread
Canthon_aequinoctialis	0.01475962	Widespread
Canthon_quinquemaculatus	0.01818762	Widespread
Canthon_cyanellus	0.0093647	Widespread
Eubucco_bourcierii	0.14074853	Widespread
Capito_aurovirens	0.1249901	Widespread
Capito_quinticolor	0.10919064	Widespread
Eubucco_richardsoni	0.09096416	Widespread
Capito_maculicoronatus	0.01275332	Widespread
Capito_squamatus	0.01337389	Widespread
Capito_auratus	0.01796758	Widespread
Capito_niger	0.15363067	Widespread
Catasticta_semiramis	0.07125222	Widespread
Catasticta_sisamnus	0.05272879	Endemic
Cathartes_aura	0.02259692	Widespread

Cathartes_burrovianus	0.02273002	Widespread
Cathartes_melambrotus	0.03421128	Widespread
Sarcoramphus_papa	0.07136998	Widespread
Vultur_gryphus	0.04386813	Widespread
Cebus_olivaceus	0.0179334	Widespread
Cebus_capucinus	0.04358278	Widespread
Cebus_albifrons	0.02617102	Widespread
Cebus_apella	0.01329163	Widespread
Saimiri_sciureus	0.01540989	Widespread
Ceroxylon_alpinum	0.00221652	Widespread
Ceroxylon_sasaimae	0.00082169	Endemic
Ceroxylon_echinulatum	0.00027438	Widespread
Ceroxylon_amazonicum	0.00027578	Endemic
Ceroxylon_parvifrons	0.00082391	Widespread
Ceroxylon_ventricosum	0.00055208	Widespread
Ceroxylon_quindiuense	0.00027542	Widespread
Ceroxylon_ceriferum	0.00110764	Widespread
Ceroxylon_vogelianum	0.00028499	Widespread
Chiroderma_salvini	0.00891815	Widespread
Chiroderma_trinitatum	0.00347192	Widespread
Chiroderma_villosum	0.00463559	Widespread
Chironius_grandisquamis	0.08016316	Widespread
Chironius_scurrulus	0.04352418	Widespread
Chironius_fuscus	0.0789436600000001	Widespread
Chironius_monticola	0.04945395	Widespread
Chironius_exoletus	0.06133661	Widespread
Chironius_carinatus	0.0308847800000001	Widespread
Chlorochrysa_phoenicotis	0.04933517	Widespread
Chlorochrysa_nitidissima	0.02957902	Widespread
Chlorochrysa_calliparaea	0.02457216	Widespread
Cissopis_leverianus	0.05469743	Widespread
Schistochlamys_melanopis	0.03920459	Widespread
Paroaria_coronata	0.03249906	Endemic
Paroaria_gularis	0.01229052	Widespread
Christensonella_uncata	0.01790415	Widespread
Christensonella_nardoides	0.01290546	Endemic
Chusquea_uniflora	2.34000000887136e-06	Widespread
Chusquea_albilanata	0.000259670000005485	Widespread
Chusquea_subulata	3.07999999904496e-06	Widespread
Chusquea_exasperata	0.000799929999999449	Endemic
Chusquea_maclurei	0.00143500000000074	Widespread
Chusquea_fendleri	0.0016563000000076	Widespread
Chusquea_purdieana	1.07999998988362e-06	Widespread
Chusquea_serpens	0.000990959999995766	Widespread

Chusquea_maculata	0.00170952000000568	Widespread
Chusquea_latifolia	0.00543510999999341	Widespread
Chusquea_spencei	0.00201778999999647	Widespread
Chusquea_perligulata	0.00101302000000203	Endemic
Chusquea_robusta	0.00104196000000911	Endemic
Chusquea_antioquensis	0.001440510000009	Widespread
Chusquea_lehmannii	0.000648580000003562	Widespread
Chusquea_subtessellata	0.00064806000000317	Widespread
Chusquea_scandens	0.000836290000009399	Widespread
Chusquea_tessellata	0.00031173000000706	Widespread
Chusquea_arachniformis	0.00236900999999534	Endemic
Chusquea_spectabilis	0.00787483999999949	Widespread
Chusquea_magnifolia	0.000456249999999159	Endemic
Chusquea_stuebelii	0.000820399999994947	Widespread
Chusquea_asymmetrica	0.00172507999999993	Endemic
Chusquea_villosa	0.000856330000004846	Endemic
Chusquea_rigida	0.000838049999998702	Endemic
Chusquea_aristata	0.00598919000000819	Widespread
Chusquea_elata	0.000183840000005375	Widespread
Chusquea_nana	0.003035440000005	Endemic
Chusquea_nobilis	0.000836919999997576	Widespread
Mycteria_americana	0.02041501	Widespread
Ciconia_maguari	0.04834408	Widespread
Columnea_rosea	0.01578853	Widespread
Columnea_dielsii	0.01308844	Widespread
Columnea_picta	0.00981809	Widespread
Columnea_medicinalis	0.02507893	Widespread
Columnea_eburnea	0.00467469	Widespread
Columnea_schimpffii	0.00480996	Widespread
Columnea_albiflora	0.00256153000000001	Widespread
Columnea_villosissima	4.29999999995712e-07	Widespread
Columnea_ericae	0.00208456	Widespread
Columnea_guttata	0.00485073999999999	Widespread
Columnea_inaequilatera	2.8200000000006e-06	Widespread
Columnea_tenella	0.01620723	Widespread
Columnea_atahualpae	0.00190712000000001	Endemic
Columnea_isernii	0.00132649	Widespread
Columnea_lophophora	0.00142848999999999	Endemic
Columnea_gloriosa	0.01432269	Widespread
Columnea_eubracteata	0.00401419	Widespread
Columnea_manabiana	0.01112263	Widespread
Columnea_tandapiana	0.05353195	Widespread
Columnea_dressleri	0.00128693000000001	Endemic
Columnea_moesta	0.00480016	Endemic

Columnea_paramicola	0.00688712999999999	Endemic
Columnea_tenensis	0.000761949999999997	Widespread
Columnea_brenneri	0.00400006	Widespread
Columnea_elongatifolia	8.60000000005301e-07	Widespread
Columnea_crassicaulis	0.00601468999999999	Endemic
Columnea_katzensteiniae	0.00444561	Widespread
Columnea_rileyi	0.00875073	Widespread
Columnea_lehmannii	8.60000000005301e-07	Widespread
Columnea_spathulata	0.00675942	Widespread
Columnea_angustata	0.00147543	Widespread
Columnea_nicaraguensis	0.00103613000000001	Widespread
Columnea_orientandina	0.00532212999999999	Widespread
Columnea_ovatifolia	0.00594759	Widespread
Columnea_colombiana	0.00380312000000001	Widespread
Columnea_kalbreyeriana	0.03046348	Widespread
Columnea_byrsina	0.01596716	Widespread
Columnea_minor	0.00444751	Widespread
Columnea_scandens	0.00668778	Widespread
Columnea_billbergiana	0.000905600000000006	Widespread
Columnea_bilabiata	0.00292269000000001	Widespread
Columnea_ciliata	0.00341512999999999	Widespread
Columnea_purpurata	8.59999999991423e-07	Widespread
Columnea_purpureovittata	0.00488330000000001	Endemic
Columnea_dissimilis	0.00785973999999999	Widespread
Columnea_fimbricalyx	0.00456508	Widespread
Columnea_rubriacuta	0.01014673	Widespread
Columnea_rubricalyx	0.0126198	Widespread
Columnea_minutiflora	0.00971459999999999	Widespread
Columnea_herthae	0.00757824999999999	Widespread
Columnea_consanguinea	0.01244609	Widespread
Columnea_sanguinea	0.00408828	Widespread
Columnea_filifera	0.00338053000000001	Widespread
Columnea_citriflora	0.00533411	Endemic
Columnea_anisophylla	0.00763905000000001	Widespread
Columnea_calotricha	0.00461729999999999	Widespread
Columnea_mira	0.00419112999999999	Endemic
Columnea_strigosa	0.00838053	Widespread
Columnea_kucyniakii	0.01136055	Widespread
Cyathea_parvula	0.00627724	Widespread
Cyathea_poeppigii	0.00210372	Widespread
Cyathea_tryonorum	0.00246986	Widespread
Cyathea_bicrenata	1.029000000001e-05	Widespread
Cyathea_karsteniana	0.000759389999999999	Endemic
Cyathea_horrida	0.000479979999999991	Widespread

Cyathea_mutica	0.000854450000000007	Widespread
Cyathea_microdonta	0.00209464000000001	Widespread
Cyathea_gibbosa	0.00112722	Endemic
Cyathea_senilis	1.20000000006226e-07	Widespread
Cyathea_pungens	0.00421250000000001	Widespread
Cyathea_andina	0.00112010999999999	Widespread
Cyathea_nigripes	0.00113759000000001	Widespread
Cyathea_lasiosora	0.00114452000000001	Widespread
Cyathea_petiolata	0.00155682	Widespread
Cyathea_multiflora	0.000261469999999986	Widespread
Cyathea_suprastrigosa	0.00106963	Widespread
Cyathea_planadae	0.00175178000000001	Widespread
Cyathea_guentheriana	0.00146210999999999	Endemic
Cyathea_schiedeana	0.000133169999999988	Widespread
Cyathea_mucilagina	0.000161919999999996	Widespread
Cyathea_gracilis	9.70000000000137e-06	Widespread
Cyathea_caracasana	0.000330259999999999	Widespread
Cyathea_fulva	0.000832949999999999	Widespread
Cyathea_divergens	1.40000000009577e-07	Widespread
Deltochilum_carinatum	0.07217439	Widespread
Deltochilum_mexicanum	0.02616402	Widespread
Deltochilum_gibbosum	0.04137776	Widespread
Colostethus_pratti	0.0685966	Widespread
Colostethus_panamansis	0.07020156	Endemic
Ameerega_bilinguis	0.05643972	Endemic
Ameerega_parvula	0.0505898699999999	Widespread
Colostethus_fraterdanieli	0.11584806	Widespread
Epipedobates_boulengeri	0.0203202	Widespread
Epipedobates_machalilla	0.01653794	Widespread
Hyloxalus_vertebralis	0.13377864	Widespread
Hyloxalus_maculosus	0.00660622999999994	Endemic
Hyloxalus_bocagei	0.0411261900000001	Widespread
Hyloxalus_sauli	0.0695938	Widespread
Hyloxalus_elachyhistus	0.10089247	Endemic
Hyloxalus_toachi	0.02419271	Endemic
Hyloxalus_awa	0.01494949	Widespread
Hyloxalus_subpunctatus	0.11831504	Widespread
Hyloxalus_delatorreae	0.14944585	Widespread
Phyllobates_aurotaenia	0.0304281900000001	Widespread
Phyllobates_terribilis	0.03507321	Endemic
Andinobates_minutus	0.0707263899999999	Widespread
Andinobates_fulguritus	0.07371558	Widespread
Oophaga_lehmanni	0.02651092	Endemic
Dendrobates_truncatus	0.0510571799999999	Widespread

Dendrobates_auratus	0.0934037	Widespread
Adelphobates_quinquevittatus	0.0876288200000001	Widespread
Phyllobates_bicolor	0.2617491	Widespread
Ameerega_hahneli	0.11525649	Widespread
Epipedobates_tricolor	0.0942432	Endemic
Andinobates_bombetes	0.00912060999999997	Widespread
Andinobates_virolinensis	0.00575048	Endemic
Andinobates_cassidyhornae	0.01307657	Endemic
Andinobates_opisthomelas	0.0144966600000001	Widespread
Ranitomeya_ventrimaculata	0.0371323600000001	Widespread
Oophaga_histrionica	0.01427832	Widespread
Veniliornis_fumigatus	0.0318924	Widespread
Veniliornis_kirkii	0.04201002	Widespread
Veniliornis_passerinus	0.00214611	Widespread
Veniliornis_frontalis	0.00264861	Endemic
Veniliornis_chocoensis	52.18454493	Widespread
Veniliornis_callonotus	0.01646037	Widespread
Veniliornis_dignus	0.02143528	Widespread
Veniliornis_nigriceps	0.02427113	Widespread
Veniliornis_affinis	0.02928182	Widespread
Sphyrapicus_varius	0.01653307	Widespread
Melanerpes_formicivorus	0.05313465	Widespread
Melanerpes_pucherani	0.05341367	Widespread
Diphylla_ecaudata	0.08597274	Widespread
Desmodus_rotundus	0.04312338	Widespread
Diaemus_youngi	0.12975758	Endemic
Caluromys_philander	0.05190029	Endemic
Caluromys_derbianus	0.06054236	Widespread
Caluromys_lanatus	0.04379881	Widespread
Marmosa_rubra	0.18215873	Widespread
Marmosa_xerophila	0.04892872	Widespread
Marmosa_robinsoni	0.05504831	Widespread
Marmosa_mexicana	0.08129581	Endemic
Marmosa_lepida	0.08243072	Endemic
Marmosa_murina	0.04538912	Widespread
Micoureus_alstoni	0.07802015	Endemic
Micoureus_demerarae	0.03635604	Widespread
Micoureus_regina	0.02224152	Widespread
Monodelphis_palliolata	0.024969	Widespread
Metachirus_nudicaudatus	0.19506084	Widespread
Chironectes_minimus	0.08278057	Widespread
Philander_frenatus	0.0610229	Widespread
Didelphis_virginiana	0.06943673	Endemic
Didelphis_albiventris	0.01592479	Widespread

Didelphis_imperfecta	0.02597232	Widespread
Didelphis_marsupialis	0.01665572	Widespread
Philander_andersoni	0.03671132	Widespread
Philander_opossum	0.02395776	Widespread
Gracilinanus_marica	0.07465685	Endemic
Gracilinanus_agilis	0.05994844	Endemic
Marmosops_impavidus	0.01859951	Widespread
Marmosops_noctivagus	0.0372628699999999	Widespread
Marmosops_parvidens	0.0257183	Widespread
Marmosops_handleyi	0.07378721	Endemic
Marmosops_invictus	0.09376184	Endemic
Marmosops_fuscatus	0.0371403100000001	Widespread
Diplazium_plantaginifolium	0.00844161	Widespread
Diplazium_bombonasae	0.00786836	Widespread
Diplazium_unilobum	0.00564502	Endemic
Diplazium_cristatum	0.00197913	Widespread
Diplazium_expansum	0.00181255	Widespread
Diplazium_megaphyllum	0.00270088	Endemic
Diplazium_pinnatifidum	0.0028441	Widespread
Diplazium_lindbergii	0.00243906000000001	Widespread
Diplazium_striatum	0.00415098000000001	Widespread
Diplazium_remotum	0.00111421	Widespread
Diplazium_macrophyllum	3.69999999999537e-07	Widespread
Diplazium_bicolor	0.00221838999999999	Endemic
Diplazium_cuneifolium	0.00326073	Endemic
Diplazium_diplazioides	1.17199999999928e-05	Widespread
Diplazium_grandifolium	0.01133912	Widespread
Diplazium_divergens	0.00114537000000001	Endemic
Diplazium_hians	0.00145766	Widespread
Diplazium_centripetale	0.00402224999999999	Endemic
Diplazium_subserratum	0.00533831	Widespread
Dipsas_indica	0.035249	Widespread
Ninia_atrata	0.0456551	Widespread
Sibon_nebulatus	0.01616738	Widespread
Dipsas_articulata	0.00459972	Widespread
Dipsas_catesbyi	0.06325504	Widespread
Dipsas_variegata	0.00181952	Widespread
Dipsas_pratti	0.00041973	Widespread
Sigesbeckia_jorullensis	0.02270386	Widespread
Sigesbeckia_orientalis	1.5999999999905e-06	Endemic
Smallanthus_pyramidalis	0.00991912	Widespread
Smallanthus_microcephalus	0.00935934999999999	Widespread
Smallanthus_riparius	1.31000000000436e-06	Widespread
Smallanthus_siegesbeckius	0.00501078999999999	Endemic

Smallanthus_fruticosus	1.0420000000011e-05	Widespread
Libanothamnus_occultus	0.0244754	Widespread
Carramboa_trujillensis	0.03715956	Endemic
Coespeletia_timotensis	0.00945135	Endemic
Espeletiopsis_pannosa	1.31000000000436e-06	Endemic
Ruilopezia_ruizii	0.01764959	Endemic
Ruilopezia_marcescens	0.00241916	Widespread
Ruilopezia_atropurpurea	0.00757695	Endemic
Tamania_chardonii	0.02010066	Widespread
Espeletia_schultzii	5.25000000001219e-06	Endemic
Coespeletia_moritziana	0.00990958	Endemic
Espeletia_pycnophylla	0.00494057000000001	Widespread
Espeletiopsis_jimenez-quesadae	5.25000000001219e-06	Widespread
Paramiflos_glandulosus	0.00246732999999999	Endemic
Ichthyothere_scandens	0.07342266	Widespread
Ichthyothere_terminalis	0	Widespread
Eulaema_sororia	0.01441385	Endemic
Eulaema_bombiformis	0.00572847999999998	Endemic
Eulaema_meriana	0.00164631999999998	Widespread
Eulaema_speciosa	0.23636622	Endemic
Eulaema_polychroma	0.02378798	Widespread
Eulaema_boliviensis	0.00603517999999997	Endemic
Eulaema_pseudocingulata	9.99999999973245e-07	Endemic
Eulaema_cingulata	9.99999999973245e-07	Widespread
Eulaema_nigrita	0.01110649	Widespread
Exaerete_smaragdina	0.01830557	Endemic
Exaerete_frontalis	0.02644544	Endemic
Euglossa_tridentata	0.01794104	Widespread
Euglossa_cognata	0.01008035	Endemic
Euglossa_mixta	0.02544611	Widespread
Euglossa_maculilabris	0.01815408	Widespread
Euglossa_cybelia	0.01494867	Widespread
Euglossa_hemichlora	0.0104276	Endemic
Euglossa_cordata	0.00613493999999998	Endemic
Euglossa_chlorina	0.00593071000000001	Endemic
Euglossa_deceptrix	0.00598568999999999	Widespread
Euglossa_variabilis	0.01052508	Endemic
Euglossa_despecta	0.00351340999999999	Endemic
Euglossa_modestior	0.01827516	Endemic
Euglossa_townsendi	0.02114823	Endemic
Euglossa_amazonica	0.00263273000000003	Widespread
Euglossa_heterosticta	0.00734516000000002	Endemic
Euglossa_hansoni	0.0119854	Endemic
Euglossa_macrorhyncha	0.01183596	Widespread

Euglossa_turbinifex	0.01313105	Endemic
Euglossa_allosticta	0.03056453	Widespread
Euglossa_ignita	0.01158231	Endemic
Euglossa_orellana	0.0083879	Widespread
Euglossa_fuscifrons	0.01540648	Endemic
Euglossa_samperi	0.01641499	Widespread
Eufriesea_pulchra	0.008969	Endemic
Eufriesea_lucifera	0.02416434	Endemic
Eufriesea_surinamensis	0.01357377	Endemic
Eufriesea_chrysopyga	0.01137105	Endemic
Eufriesea_magrettii	0.01060753	Endemic
Falco_sparverius	0.03944703	Widespread
Falco_columbarius	0.01436407	Widespread
Falco_deiroleucus	0.00792826000000001	Widespread
Falco_femoralis	0.00912967000000001	Widespread
Falco_peregrinus	0.00531739000000001	Widespread
Falco_rufigularis	0.00074247999999999	Widespread
Panthera_onca	0.00177908	Widespread
Puma_concolor	0.00178741	Widespread
Puma_yagouaroundi	0.00228621	Widespread
Leopardus_colocolo	0.000803679999999999	Endemic
Leopardus_tigrinus	0.000589009999999999	Widespread
Leopardus_wiedii	0.00117308	Widespread
Leopardus_pardalis	0.0032398	Widespread
Fregata_magnificens	3.97000000001979e-06	Widespread
Geonoma_deversa	0.00112527	Widespread
Geonoma_interrupta	0.0008236	Widespread
Geonoma_pinnatifrons	0.00032201	Widespread
Geonoma_frontinensis	0.0021578	Widespread
Geonoma_poeppigiana	0.00208436	Endemic
Geonoma_brongniartii	0.00094585	Widespread
Geonoma_longepedunculata	0.00285594	Widespread
Geonoma_hollinensis	0.00213573	Widespread
Geonoma_divisa	0.00272581	Widespread
Geonoma_triandra	0.00146369	Widespread
Geonoma_ferruginea	0.00018591	Endemic
Geonoma_orbignyana	0.00125921	Widespread
Geonoma_lehmannii	0.00217389	Widespread
Geonoma_undata	0.00050834	Widespread
Geonoma_tenuissima	0.00096142	Widespread
Geonoma_cuneata	0.00177457	Widespread
Geonoma_stricta	0.00241424	Widespread
Geonoma_leptospadix	0.00112185	Widespread
Geonoma_lanata	0.00179676	Widespread

Geonoma_bernalii	0.00098148	Endemic
Geonoma_simplicifrons	0.00247975	Widespread
Geonoma_pauciflora	0.00103193	Endemic
Geonoma_pohliana	0.00082196	Endemic
Geonoma_maxima	0.00353157	Widespread
Geonoma_congesta	0.00187684	Widespread
Geonoma_calyptrogynoidea	0.0011972	Widespread
Geonoma_triglochin	4.30000000000048e-07	Widespread
Geonoma_concinna	0.0004935	Widespread
Geonoma_multisecta	0.00180768	Widespread
Geonoma_camana	0.00284801	Endemic
Geonoma_macrostachys	0.00117895	Widespread
Choeroniscus_godmani	0.01604902	Widespread
Choeroniscus_minor	0.05443067	Widespread
Lichonycteris_obscura	0.05600419	Widespread
Hylonycteris_underwoodi	0.15117513	Endemic
Anoura_cultrata	0.06468379	Widespread
Anoura_caudifer	0.08889086	Widespread
Anoura_geoffroyi	0.04751273	Widespread
Anoura_latidens	0.06179874	Widespread
Glossophaga_soricina	0.09991896	Widespread
Glossophaga_commissarisi	0.0894681899999999	Widespread
Glossophaga_longirostris	0.05022757	Widespread
Glossophaga_leachii	0.05375237	Endemic
Leptonycteris_curasoae	0.00980146999999998	Widespread
Lonchophylla_mordax	0.00364041999999998	Widespread
Lonchophylla_concava	0.000958930000000024	Widespread
Lionycteris_spurrelli	0.1258598	Widespread
Lonchophylla_chocoana	0.0890497	Widespread
Lonchophylla_robusta	0.07037846	Widespread
Lonchophylla_handleyi	0.06913009	Endemic
Gnetum_leyboldii	0.00960796000000003	Widespread
Gnetum_urens	0.01543247	Widespread
Gnetum_nodiflorum	0.00421318999999998	Widespread
Gnetum_schwackeanum	0.01225314	Widespread
Gonatodes_albogularis	0.15891727	Widespread
Gonatodes_vittatus	0.15730273	Widespread
Gonatodes_ocellatus	0.05394355	Endemic
Gonatodes_concinnatus	0.19957701	Widespread
Gonatodes_caudiscutatus	0.29295441	Widespread
Lepidoblepharis_xanthostigma	0.31515634	End
Lepidoblepharis_festae	0.25446895	Endemic
Lepidoblepharis_sanctaemartae	0.50742884	Widespread
Habenaria_obtusa	0.01840438	Widespread

Habenaria_distans	0.01345369	Endemic
Habenaria_monorrhiza	0.01909618	Widespread
Habenaria_longicauda	0.00518373999999999	Widespread
Habenaria_macronectar	1.5999999999905e-06	Endemic
Habenaria_bractescens	0.00128342000000001	Endemic
Habenaria_repens	0.00658048	Widespread
Habenaria_trifida	0.01059863	Widespread
Habenaria_armata	0.00233533	Widespread
Habenaria_alata	0.00230183	Widespread
Habenaria_caldensis	0.01056272	Endemic
Habenaria_rodeiensis	0.01250524	Endemic
Habenaria_laevigata	0.03663466	Widespread
Heliconius_aliphera	0.04979113	Widespread
Heliconius_melpomene	0.0146759	Widespread
Heliconius_timareta	0.01854419	Endemic
Heliconius_cydno	0.21298894	Widespread
Heliconius_heurippa	0.00259317999999997	Endemic
Heliconius_ismenius	0.02762058	Widespread
Heliconius_ethilla	0.01653835	Widespread
Heliconius_godmani	0.12380788	Endemic
Heliconius_hecale	0.01155142	Widespread
Heliconius_hecuba	0.03430836	Endemic
Heliconius_xanthocles	0.04639259	Endemic
Heliconius_doris	0.05212785	Widespread
Heliconius_wallacei	0.28303788	Endemic
Heliconius_sara	0.02282561	Widespread
Heliconius_leucadia	0.04433206	Endemic
Heliconius_antiochus	0.03220506	Widespread
Heliconius_sapho	0.03722455	Widespread
Heliconius_eleuchia	0.02743403	Widespread
Heliconius_charithonia	0.02017228	Widespread
Heliconius_hecalesia	0.04266173	Widespread
Heliconius_erato	0.0162662	Widespread
Heliconius_clysonymus	0.0251268100000001	Widespread
Heliconius_telesiphe	0.0431754600000001	Widespread
Agalychnis_spurrelli	0.02836996	Widespread
Phyllomedusa_tomopterna	0.20573916	Widespread
Trachycephalus_venulosus	0.00895975999999998	Widespread
Osteocephalus_buckleyi	0.04944469	Widespread
Smilisca_sila	0.0601292	Widespread
Smilisca_phaeota	0.04872511	Widespread
Hyla_molleri	0.03153018	Endemic
Hypsiboas_punctatus	0.0748487	Widespread
Hypsiboas_lanciformis	0.0660595	Widespread

Hypsiboas_pugnax	0.04711167	Widespread
Hypsiboas_crepitans	0.03936426	Widespread
Hypsiboas_rosenbergi	0.04595224	Widespread
Hypsiboas_boans	0.14394769	Widespread
Hyloscirtus_colymba	0.06550661	Endemic
Hyloscirtus_alytolylax	0.01753004	Widespread
Hyloscirtus_phyllognathus	0.0751415999999999	Widespread
Hyloscirtus_lascinius	0.06334301	Endemic
Hyloscirtus_palmeri	0.07291143	Widespread
Hyloscirtus_psarolaimus	0.02485221	Widespread
Hyloscirtus_staufferorum	0.01251238	Endemic
Hyloscirtus_ptychodactylus	0.00587503	Endemic
Hyloscirtus_lindae	0.00802547999999997	Widespread
Hyloscirtus_pantostictus	0.00599240000000001	Endemic
Dendropsophus_carnifex	0.0731467	Widespread
Sphaenorhynchus_lacteus	0.04406091	Endemic
Osteocephalus_verruciger	0.01295744	Widespread
Osteocephalus_cabrerai	0.0148677500000001	Endemic
Osteocephalus_mutabor	0.00217491999999997	Widespread
Osteocephalus_carri	0.12621863	Endemic
Osteocephalus_alboguttatus	0.04234863	Widespread
Osteocephalus_taurinus	0.02274814	Widespread
Trachycephalus_jordani	0.24668046	Widespread
Dendropsophus_parviceps	0.0601531	Widespread
Hypsiboas_picturatus	0.07717717	Widespread
Hypsiboas_pellucens	0.01783309	Widespread
Hypsiboas_rufitelus	0.02734521	Endemic
Scinax_boesemani	0.0847101100000001	Endemic
Dendropsophus_meridensis	0.00672291999999997	Widespread
Dendropsophus_labialis	2.57999999997427e-06	Widespread
Scinax_sugillatus	0.13453273	Widespread
Osteocephalus_planiceps	0.0109744900000001	Endemic
Osteocephalus_fuscifacies	0.02305136	Endemic
Osteocephalus_deridens	0.03240562	Endemic
Osteocephalus_leprieurii	0.00819331000000001	Widespread
Pseudis_paradoxa	0.07374902	Widespread
Scinax_acuminatus	0.04971041	Endemic
Scinax_quinquefasciatus	0.06805941	Widespread
Scinax_boulengeri	0.0580667600000001	Widespread
Scinax_rostratus	0.0292983	Widespread
Scinax_kennedyi	0.02232013	Endemic
Scinax_elaeochrous	0.0559778	Widespread
Scinax_cruentommus	0.07661281	Widespread
Scinax_wandae	0.06475641	Widespread

Scinax_funereus	0.05692226	Widespread
Scinax_ruber	0.05595849	Widespread
Dendropsophus_ebraccatus	0.04795994	Widespread
Dendropsophus_subocularis	0.0262814	Widespread
Dendropsophus_triangulum	0.03465232	Widespread
Dendropsophus_leucophyllatus	0.03292621	Widespread
Dendropsophus_sarayacuensis	0.03666411	Widespread
Dendropsophus_bifurcus	0.05296542	Widespread
Dendropsophus_riveroi	0.0591027000000001	Endemic
Dendropsophus_minusculus	0.02361571	Endemic
Dendropsophus_mathiassoni	0.01134336	Widespread
Dendropsophus_microcephalus	0.0575475599999999	Widespread
Dendropsophus_phlebodes	0.0356396999999999	Widespread
Dendropsophus_rhodopeplus	0.04458103	Widespread
Dendropsophus_bokermanni	0.07230824	Widespread
Dendrobates_truncatus	0.0892605100000001	Widespread
Dendropsophus_marmoratus	0.05400977	Widespread
Cruziohyla_calcarifer	0.0554110800000001	Endemic
Hylomantis_hulli	0.03420959	Endemic
Phyllomedusa_perinesos	0.0502792200000001	Widespread
Phyllomedusa_hypochondrialis	0.15700147	Widespread
Phyllomedusa_bicolor	0.0597600899999999	Widespread
Kinosternon_leucostomum	0.12953025	Widespread
Kinosternon_scorpioides	0.02384345	Widespread
Kinosternon_dunni	0.01834405	Endemic
Nothopsis_rugosus	0.16875524	Widespread
Leptodeira_annulata	0.07738018	Widespread
Leptodeira_septentrionalis	0.03499712	Widespread
Imantodes_cenchoa	0.0384621	Widespread
Imantodes_inornatus	0.04484109	Widespread
Imantodes_gemmistratus	0.11950411	Endemic
Imantodes_chocoensis	0.05320079	Endemic
Imantodes_lentiferus	0.07006995	Widespread
Lupinus_pubescens	3.79999999994274e-07	Widespread
Lupinus_prostratus	0.000494710000000009	Endemic
Lupinus_nubigenus	0.000987070000000007	Widespread
Lupinus_solanagrorum	7.50000000007689e-07	Endemic
Lupinus_sarmentosus	2.00000000033507e-08	Widespread
Lupinus_ramosissimus	1.89999999997137e-07	Widespread
Lupinus_huigrensis	1.48300000000073e-05	Endemic
Lupinus_arvensis	0	Widespread
Lupinus_microphyllus	0.00049115000000001	Widespread
Lupinus_lindleyanus	0.00147643	Endemic
Lupinus_perennis	9.99999999473644e-09	Endemic

Lupinus_guaraniticus	0.00399408	Endemic
Lupinus_semperflorens	0.00221503000000001	Widespread
Lupinus_bogotensis	3.79999999994274e-07	Widespread
Lupinus_mutabilis	1.89999999983259e-07	Widespread
Lupinus_albus	0.00842013999999999	Widespread
Lontra_longicaudis	0.00158462	Widespread
Pteronura_brasiliensis	0.00386959	Widespread
Cerdocyon_thous	0.01465211	Widespread
Phlegmariurus_lindenii	0.00271725	Widespread
Phlegmariurus_campianus	0.00248598	Widespread
Phlegmariurus_rosenstockianus	0.00236598999999998	Widespread
Phlegmariurus_linifolius	0.000886979999999982	Widespread
Phlegmariurus_sarmentosus	0.000442780000000004	Widespread
Phlegmariurus_reflexus	0.000845579999999985	Widespread
Phlegmariurus_eversus	4.29999999995712e-07	Widespread
Huperzia_eversa	4.29999999995712e-07	Widespread
Phlegmariurus_capellae	4.29999999995712e-07	Widespread
Phlegmariurus_attenuatus	0.000599070000000007	Widespread
Phlegmariurus_crassus	0.001106	Widespread
Phlegmariurus_cumingii	0.00124759000000002	Widespread
Phlegmariurus_hystrix	4.29999999995712e-07	Widespread
Phlegmariurus_rufescens	1.5999999999905e-07	Widespread
Phlegmariurus_compactus	0.00107305999999999	Endemic
Phlegmariurus_dichotomus	0.00486803000000002	Widespread
Phlegmariurus_hippurideus	0.00115379000000002	Widespread
Huperzia_wilsonii	0.00287406000000001	Endemic
Phlegmariurus_dichaeoides	0.02737332	Widespread
Phlegmariurus_ericifolius	4.29999999995712e-07	Widespread
Huperzia_selago	0.000550379999999989	Endemic
Huperzia_miyoshiana	2.10000000000488e-07	Endemic
Lycopodium_jussiaei	0.00942492	Widespread
Diphasiastrum_complanatum	4.65999999998967e-06	Widespread
Diphasiastrum_thyoides	0.01202295	Widespread
Lycopodium_vestitum	3.4780000000012e-05	Endemic
Lycopodium_clavatum	0.01130376	Widespread
Lycopodiella_alopecuroides	0.00498477999999999	Widespread
Phlegmariurus_funiformis	0.00408743	Widespread
Phlegmariurus_filiformis	0.00427547	Endemic
Macleania_coccoloboides	0.00374889	Widespread
Macleania_bullata	0.00448419	Widespread
Macleania_floribunda	0.00277325	Widespread
Macleania_cordifolia	0.00392019	Widespread
Macleania_rupestris	0.0039251	Widespread
Macleania_insignis	0.00235672	Endemic

Macleania_rotundifolia	0.01590158	Widespread
Macleania_smithiana	0.00134869	Widespread
Androdon_aequatorialis	0.02767055	Widespread
Polytmus_guainumbi	0.01142814	Widespread
Polytmus_milleri	0.00703247	Endemic
Chrysolampis_mosquitus	0.01075025	Widespread
Anthracothorax_nigricollis	0.00473622	Widespread
Anthracothorax_prevostii	0.03080905	Widespread
Schistes_geoffroyi	0.01770383	Widespread
Doryfera_johannae	0.00957584	Widespread
Colibri_thalassinus	0.00997312	Widespread
Colibri_coruscans	0.00833766	Widespread
Colibri_delphinae	0.00935633	Widespread
Drymoluber_dichrous	0.04262663	Widespread
Mastigodryas_melanolomus	0.05598571	Endemic
Mastigodryas_boddaerti	0.03143241	Widespread
Mastigodryas_bifossatus	0.02267653	Widespread
Maxillaria_suarezorum	0.03023372	Endemic
Maxillaria_gigantea	0.02054001	Widespread
Maxillaria_splendens	0.03194021	Widespread
Maxillaria_bradei	0.00512545	Widespread
Maxillaria_bolivarensis	0.00858863	Widespread
Maxillaria_ochroleuca	0.00631215	Widespread
Maxillaria_pulla	0.01457046	Endemic
Maxillaria_chionantha	0.01179619	Endemic
Maxillaria_brachybulbon	0.02043693	Widespread
Maxillaria_fractiflexa	0.00615847999999999	Widespread
Maxillaria_lepidota	0.01134324	Widespread
Maxillaria_whittenii	0.01701991	Endemic
Maxillaria_neophylla	0.00395848	Widespread
Maxillaria_rubioi	0.00288589	Widespread
Maxillaria_porrecta	0.00579715	Widespread
Maxillaria_cryptobulbon	0.01071749	Widespread
Maxillaria_ringens	0.00612190999999999	Widespread
Maxillaria_perryae	0.0086232	Endemic
Maxillaria_pauciflora	0.01028025	Endemic
Maxillaria_angustissima	0.0177039	Widespread
Maxillaria_reichenheimiana	0.00624742	Endemic
Maxillaria_atwoodiana	0.0053137	Endemic
Maxillaria_patens	0.00225936	Widespread
Maxillaria_jucunda	0.00166042	Endemic
Maxillaria_molitor	0.000552800000000006	Endemic
Maxillaria_longissima	0.00464085	Widespread
Maxillaria_striata	0.01733485	Endemic

Maxillaria_fletcheriana	0.00449098000000001	Widespread
Maxillaria_grandis	0.00168289000000001	Widespread
Maxillaria_sanderiana	0.00304159	Widespread
Maxillaria_meridensis	0.01439333	Widespread
Maxillaria_jostii	0.00616319	Endemic
Megascops_guatemalae	0.0629504600001383	Widespread
Megascops_roboratus	0.0464218300000994	Widespread
Megascops_watsonii	0.0179701000001842	Widespread
Megascops_petersoni	0.0822578699999212	Widespread
Megascops_colombianus	0.0309900600000219	Widespread
Megascops_ingens	0.0315769500000442	Widespread
Megascops_choliba	0.0604833100001088	Widespread
Megascops_albogularis	0.0568137000000206	Widespread
Melocactus_curvispinus	3.45000000000206e-06	Widespread
Conepatus_semistriatus	0.00877575999999999	Widespread
Metallura_tyrianthina	0.00715	Widespread
Chalcostigma_herrani	0.00567618	Widespread
Chalcostigma_stanleyi	0.00557961	Widespread
Oxypogon_guerinii	0.00455356	Widespread
Metallura_williami	0.000310970000000001	Widespread
Micrathena_sagittata	0.06376142	Endemic
Micrathena_sexspinosa	0.06261938	Widespread
Micrathena_plana	0.12617883	Widespread
Micrathena_horrida	0.19540592	Endemic
Micrathena_schreibersi	0.19179838	Endemic
Micrurus_psyches	0.01540443	Widespread
Micrurus_nigrocinctus	0.05080706	Widespread
Micrurus_narduccii	0.1585076	Widespread
Micrurus_hemprichii	0.08085702	Endemic
Micrurus_surinamensis	0.05686298	Widespread
Micrurus_lemniscatus	0.04692877	Widespread
Micrurus_spixii	0.02558639	Endemic
Micrurus_obscurus	0.02528187	Widespread
Micrurus_mipartitus	0.08872835	Widespread
Micrurus_dissoleucus	0.12574378	Widespread
Electron_platyrhynchum	0.06405626	Widespread
Hylomanes_momotula	0.12375633	Widespread
Baryphthengus_ruficapillus	0.02997596	Widespread
Baryphthengus_martii	0.03286451	Widespread
Momotus_momota	0.0309753	Widespread
Pteronotus_parnellii	0.14799708	Widespread
Pteronotus_davyi	0.03923782	Widespread
Pteronotus_gymnonotus	0.03491095	Widespread
Pteronotus_personatus	0.22596993	Widespread

Mormoops_megalophylla	0.19840481	Widespread
Morpho_granadensis	0.04095455	Endemic
Morpho_peleides	0.01778944	Widespread
Morpho_helenor	0.00830025000000001	Widespread
Morpho_achilles	0.02205218	Endemic
Morpho_amathonte	0.03554068	Endemic
Morpho_menelaus	0.00993886999999999	Widespread
Morpho_sulkowskyi	0.04191605	Widespread
Morpho_telemachus	0.02932245	Endemic
Morpho_theseus	0.0113113	Widespread
Morpho_cypris	0.04607192	Widespread
Galictis_vittata	0.00281016	Widespread
Galictis_cuja	0.00316152	Endemic
Mustela_frenata	0.00435829	Widespread
Cerdocyon_thous	0.021841415	Widespread
Napeogenes_sulphureophila	0.00191755	Endemic
Napeogenes_cranto	0.0133776	Endemic
Napeogenes_tolosa	0.01045352	Widespread
Napeogenes_stella	0.00384556	Widespread
Napeogenes_sylphis	0.00542795	Endemic
Napeogenes_apulia	0.00783741	Endemic
Napeogenes_inachia	0.00136756	Widespread
Napeogenes_peridia	0.00823613	Widespread
Napeogenes_glycera	0.00781934	Endemic
Napeogenes_larina	0.00874869	Endemic
Natalus_stramineus	0.0177248	Endemic
Natalus_tumidirostris	0.02106857	Widespread
Chilonatalus_tumidifrons	0.06861281	Endemic
Noctilio_leporinus	0.04119552	Widespread
Noctilio_albiventris	0.02325136	Widespread
Thyroptera_tricolor	0.15121427	Widespread
Thyroptera_lavali	0.01066399	Endemic
Thyroptera_discifera	0.04333983	Widespread
Opuntia_pittieri	1.30999999997661e-06	Widespread
Opuntia_schumannii	0.00260029000000001	Widespread
Opuntia_ficus-indica	0.00171485999999998	Widespread
Opuntia_bella	1.99999999894729e-08	Endemic
Opuntia_bakeri	0.00181472999999999	Endemic
Opuntia_caracassana	5.00000000014378e-07	Widespread
Opuntia_quitensis	0.000901929999999995	Endemic
Opuntia_helleri	0.00117738000000001	Endemic
Opuntia_pubescens	0.37439372	Widespread
Opuntia_stricta	0	Widespread
Ortalis_ruficauda	0.00161805999999998	Widespread

Ortalis_garrula	0.01289553	Widespread
Ortalis_guttata	0.0263182799999999	Widespread
Ortalis_cinereiceps	0.01608628	Widespread
Pereskia_aculeata	0.00025559	Widespread
Micronycteris_schmidtorum	0.03948477	Widespread
Micronycteris_minuta	0.02618161	Widespread
Micronycteris_microtis	0.01786506	Widespread
Micronycteris_megalotis	0.00665495999999999	Widespread
Lampronycteris_brachyotis	0.00297473999999998	Widespread
Trinycteris_nicefori	0.000270669999999973	Widespread
Glyphonycteris_sylvestris	0.09815427	Widespread
Glyphonycteris_daviesi	0.09410875	Endemic
Lonchorhina_aurita	0.099209	Widespread
Mimon_bennettii	0.03501173	Endemic
Chrotopterus_auritus	0.10930593	Widespread
Vampyrum_spectrum	0.0960620599999999	Widespread
Tonatia_bidens	0.04395703	Widespread
Tonatia_saurophila	0.04025628	Widespread
Mimon_crenulatum	0.11298541	Widespread
Phyllostomus_discolor	0.22255469	Widespread
Phyllostomus_latifolius	0.02359679	Endemic
Phyllostomus_elongatus	0.05260961	Widespread
Phyllostomus_hastatus	0.0429426	Widespread
Macrophyllum_macrophyllum	0.13761656	Widespread
Trachops_cirrhosus	0.11870195	Widespread
Piper_pseudolindenii	0.03039822	Widespread
Piper_amalago	0.0038449599999999	Widespread
Piper_reticulatum	0.0269273200000001	Widespread
Piper_darienense	0.03536231	Widespread
Piper_anisum	0.00542870000000006	Endemic
Piper_marginatum	0.03108566	Widespread
Piper_multiplinervium	0.02091343	Widespread
Piper_cararense	0.00872142999999992	Widespread
Piper_nudifolium	2.42000000005849e-06	Widespread
Piper_hispidum	0.02182894	Widespread
Piper_villiramulum	0.01681025	Widespread
Piper_immutatum	0.00923947000000003	Widespread
Piper_colonense	0.02305963	Endemic
Piper_lanceifolium	2.42000000005849e-06	Widespread
Piper_filistilum	0.01609447	Widespread
Piper_brachypodon	0.0168545099999999	Widespread
Piper_oxystachyum	0.00475980999999992	Widespread
Piper_ottoniaefolium	0.00667814999999994	Widespread
Piper_costatum	0.00252151	Widespread

Piper_divaricatum	0.00303939999999991	Widespread
Piper_crassinervium	0.04734163	Widespread
Piper_subscutatum	0.00243519999999997	Widespread
Piper_callosum	1.20999999997373e-06	Widespread
Piper_munchanum	0.00476052999999999	Widespread
Piper_augustum	1.66600000000017e-05	Widespread
Piper_garagaranum	0.0220855600000001	Widespread
Piper_trigonum	0.03401935	Widespread
Piper_arieianum	2.42000000005849e-06	Widespread
Piper_confertinodum	0.00648802999999998	Widespread
Piper_chuarense	0.00300451000000002	Widespread
Piper_longispicum	0.0117124799999999	Widespread
Piper_bellidifolium	0.00351541	Widespread
Piper_fimbriulatum	0.00603942000000002	Widespread
Piper_euryphyllum	0.00269670999999994	Endemic
Piper_maxonii	0.00278550999999994	Endemic
Piper_gibbosum	0.00475766	Endemic
Piper_obliquum	0.00355172000000004	Widespread
Piper_squamulosum	0.00771473	Widespread
Piper_daguanum	0.00979050000000004	Widespread
Piper_melanocladum	0.00382191000000009	Widespread
Piper_candollei	0.00234639999999997	Widespread
Piper_cernuum	0.00442419999999999	Widespread
Piper_truncatum	0.00624106000000002	Endemic
Piper_obtusilimbum	0.000865369999999976	Widespread
Piper_marsupiiferum	0.00371036999999996	Widespread
Piper_pulchrum	0.281844	Widespread
Piper_biseriatum	0.00172039999999996	Widespread
Piper_tuberculatum	0.05638161	Widespread
Piper_arboreum	0.01929335	Widespread
Piper_auritum	0.04363016	Widespread
Piper_peltatum	0.03808489	Widespread
Piper_umbellatum	0.0295564300000001	Widespread
Piper_cinereum	0.0651920499999999	Widespread
Callicebus_cupreus	0.0176962	Widespread
Callicebus_moloch	0.01129074	Endemic
Callicebus_torquatus	0.00188747	Widespread
Pithecia_monachus	0.00217637999999998	Endemic
Vampyrodes_caraccioli	0.10629395	Widespread
Platyrrhinus_lineatus	0.0320802	Widespread
Platyrrhinus_ismaeli	0.01249534	Widespread
Platyrrhinus_dorsalis	0.03955161	Widespread
Platyrrhinus_brachycephalus	0.08386044	Widespread
Platyrrhinus_matapalensis	0.02644683	Widespread

Platyrrhinus_helleri	0.03432528	Widespread
Platyrrhinus_vittatus	0.01178786	Widespread
Platyrrhinus_albericoi	0.01257455	Widespread
Platyrrhinus_aurarius	0.03279574	Widespread
Platyrrhinus_infuscus	0.03355175	Widespread
Uroderma_magnirostrum	0.0235235	Widespread
Uroderma_bilobatum	0.08094543	Widespread
Podilymbus_podiceps	0.06437928	Widespread
Tachybaptus_dominicus	0.05276992	Widespread
Podiceps_major	0.04643424	Endemic
Pristimantis_crenunguis	0.0806233800000022	Endemic
Pristimantis_labiosus	0.110796929999999	Widespread
Pristimantis_actites	0.215495920000002	Endemic
Pristimantis_lanthanites	0.293094889999999	Widespread
Pristimantis_caryophyllaceus	0.276856479999999	Widespread
Pristimantis_cremnobates	0.17570212	Endemic
Pristimantis_ridens	0.214326639999999	Widespread
Pristimantis_colomai	0.187398560000002	Widespread
Pristimantis_cruentus	0.1858769	Widespread
Pristimantis_erythropleura	0.0755437200000024	Widespread
Pristimantis_paisa	0.00265490000000312	Widespread
Pristimantis_viejas	0.0270029400000027	Endemic
Pristimantis_gaigei	0.199526819999999	Widespread
Pristimantis_conspicillatus	0.137087999999999	Widespread
Pristimantis_condor	0.0865333200000009	Endemic
Pristimantis_malkini	0.145136139999998	Endemic
Pristimantis_achatinus	0.112693140000001	Widespread
Pristimantis_fenestratus	0.037614640000001	Endemic
Pristimantis_savagei	0.174628169999998	Widespread
Pristimantis_nervicus	0.0807589499999999	Widespread
Pristimantis_affinis	0.0929430800000013	Widespread
Pristimantis_caprifer	0.262984169999999	Widespread
Pristimantis_eriphus	0.143160990000002	Endemic
Pristimantis_suetus	0.0987633200000033	Widespread
Pristimantis_peruvianus	0.16733962	Widespread
Pristimantis_acerus	0.105915800000002	Endemic
Pristimantis_inusitatus	0.0672706600000019	Endemic
Pristimantis_glandulosus	0.126697880000002	Endemic
Pristimantis_orcesi	0.160239950000001	Endemic
Pristimantis_appendiculatus	0.216365150000001	Endemic
Pristimantis_dissimulatus	0.11745951	Endemic
Pristimantis_calcarulatus	0.193358509999999	Endemic
Pristimantis_crucifer	0.258070780000001	Endemic
Pristimantis_subsigillatus	8.09000000145943e-06	Widespread

Pristimantis_nyctophylax	0.206873959999999	Endemic
Pristimantis_moro	0.11760044	Endemic
Pristimantis_acuminatus	0.2504314	Widespread
Pristimantis_galdi	0.146247389999999	Widespread
Pristimantis_quaquaversus	0.0816560499999994	Widespread
Pristimantis_latidiscus	25.8444508	Widespread
Pristimantis_cryophilius	0.417796880000001	Endemic
Pristimantis_phoxocephalus	0.132761080000002	Widespread
Pristimantis_riveti	0.111169199999999	Endemic
Pristimantis_versicolor	0.128341370000001	Endemic
Pristimantis_simonbolivari	0.10522027	Endemic
Pristimantis_ockendeni	0.230231710000002	Widespread
Pristimantis_unistrigatus	0.103761679999998	Widespread
Pristimantis_cajamarcensis	0.157559899999999	Endemic
Pristimantis_frater	0.13665228	Widespread
Pristimantis_bogotensis	0.104010769999999	Widespread
Pristimantis_taeniatus	0.132545889999999	Widespread
Pristimantis_miyatai	0.142139579999998	Widespread
Pristimantis_zophus	0.10333821	Widespread
Pristimantis_martiae	0.124064820000001	Widespread
Pristimantis_librarius	0.160034470000003	Widespread
Pristimantis_parvillus	0.171021679999999	Widespread
Pristimantis_luteolateralis	0.0423974499999993	Widespread
Pristimantis_walkeri	0.0352129699999999	Widespread
Pristimantis_chalceus	0.202495280000001	Widespread
Pristimantis_croceoinguinis	0.134114950000001	Widespread
Pristimantis_diadematus	0.161574880000003	Widespread
Pristimantis_altamazonicus	0.10820842	Widespread
Pristimantis_brevifrons	0.19409856	Widespread
Pristimantis_leoni	0.11940959	Widespread
Pristimantis_pyrrhomerus	0.106819999999999	Endemic
Pristimantis_ocreatus	0.1100669	Endemic
Pristimantis_celator	0.153780299999998	Endemic
Pristimantis_verecundus	0.237887690000001	Widespread
Pristimantis_thymalopsoides	0.15419078	Endemic
Pristimantis_duellmani	0.136927840000002	Endemic
Pristimantis_vertebralis	0.123542350000001	Endemic
Pristimantis_curtipes	2.72999999850754e-06	Widespread
Pristimantis_buckleyi	2.72999999850754e-06	Widespread
Pristimantis_devillei	0.113414799999997	Widespread
Pristimantis_surdus	0.113324739999999	Endemic
Pristimantis_truebae	0.0117903300000002	Endemic
Pristimantis_gentryi	0.0226633400000011	Endemic
Pristimantis_thymelensis	0.063890139999998	Endemic

Pristimantis_chloronotus	0.10762471	Widespread
Pristimantis_supernatis	0.123622600000001	Widespread
Potos_flavus	0.04576388	Widespread
Procyon_lotor	0.09734753	Endemic
Procyon_cancrivorus	0.01417711	Widespread
Nasua_nasua	0.0222946	Widespread
Nasua_narica	0.01329512	Widespread
Nasuella_olivacea	0.01408321	Widespread
Bassaricyon_neblina	0.01716377	Widespread
Bassaricyon_gabbii	0.00925985	Widespread
Bassaricyon_alleni	0.00668605999999999	Widespread
Aulacorhynchus_prasinus	0.0816906	Widespread
Aulacorhynchus_haematopygus	0.04413739	Widespread
Aulacorhynchus_sulcatus	0.04661797	Widespread
Aulacorhynchus_derbianus	0.03963737	Widespread
Selenidera_spectabilis	0.06960659	Widespread
Andigena_hypoglauca	0.0218924	Widespread
Andigena_laminirostris	0.03468031	Widespread
Andigena_nigrirostris	0.02764129	Widespread
Selenidera_reinwardtii	0.03486737	Widespread
Pteroglossus_castanotis	0.02619174	Widespread
Pteroglossus_pluricinctus	0.0115172099999999	Widespread
Pteroglossus_torquatus	0.05390933	Widespread
Pteroglossus_aracari	0.03093337	Endemic
Pteroglossus_viridis	0.03130407	Endemic
Pteroglossus_inscriptus	0.02815117	Widespread
Ramphastos_sulfuratus	0.0509039	Widespread
Ramphastos_vitellinus	0.02421481	Widespread
Ramphastos_brevis	0.01831779	Widespread
Ramphastos_tucanus	0.02663549	Widespread
Ramphastos_swainsonii	0.00836088000000001	Widespread
Ramphastos_ambiguus	0.01084865	Widespread
Sphagnum_palustre	3.6420000000037e-05	Endemic
Sphagnum_magellanicum	0.000927800000000034	Widespread
Sphagnum_perichaetiale	1.03299999999473e-05	Widespread
Sphagnum_cyclophyllum	0.07232875	Widespread
Sphagnum_sonsonense	0.000865919999999964	Widespread
Sphagnum_cuculliforme	0.00225471999999993	Endemic
Sphagnum_strictum	0.0166913599999999	Widespread
Sphagnum_recurvum	0.000626030000000055	Widespread
Sphagnum_tenellum	0.00460410999999994	Widespread
Sphagnum_cuspidatum	0.00049005000000002	Widespread
Sphagnum_sancto-josephense	0.00106340999999999	Widespread
Sphagnum_pulchrum	0.000556879999999982	Endemic

Sphagnum_laxirameum	0.000612959999999996	Endemic
Sphagnum_aciphyllum	0.000817469999999987	Endemic
Sphagnum_subsecundum	0.00505357000000006	Widespread
Sphagnum_flaccidum	0.00208454000000002	Endemic
Sphagnum_santanderense	0.00550921999999998	Endemic
Sphagnum_lescurii	0.00215348999999998	Widespread
Sphagnum_rotundatum	0.00100794999999998	Widespread
Sphagnum_denticulatum	0.00165735	Widespread
Sphagnum_platyphyllum	0.01084043	Endemic
Sphagnum_laegaardii	0.00848948000000005	Endemic
Sphagnum_compactum	0.000877530000000015	Widespread
Sphagnum_oxyphyllum	0.00158216999999994	Widespread
Sphagnum_squarrosum	0.00729684999999997	Endemic
Sphagnum_capillifolium	2.21000000000249e-06	Widespread
Sphagnum_tenerum	0.00579854000000002	Widespread
Sphagnum_nemoreum	0.00426212999999998	Widespread
Sphagnum_sparsum	1.00000000502476e-08	Widespread
Sphagnum_limbatum	0.000351419999999991	Widespread
Sphagnum_meridense	0.00213198000000003	Widespread
Sphagnum_lewisii	0.000581730000000058	Endemic
Sphagnum_austro-americanum	0.000000000000000001	Endemic
Sturnira_bidens	0.0655008	Widespread
Sturnira_lilium	0.01621833	Widespread
Sturnira_luisi	0.01775273	Widespread
Sturnira_tildae	0.04554854	Widespread
Sturnira_mordax	0.03092379	Widespread
Sturnira_magna	0.0363642	Widespread
Sturnira_erythromos	0.03406201	Widespread
Sturnira_bogotensis	0.03053041	Widespread
Sturnira_ludovici	0.0283377	Widespread
Sturnira_oporaphilum	1.60999999998523e-06	Widespread
Tangara_palmeri	0.09790858	Widespread
Tangara_punctata	0.04886694	Widespread
Tangara_guttata	0.03737545	Widespread
Tangara_xanthogastra	0.0351014	Widespread
Tangara_larvata	0.02551348	Widespread
Tangara_cyanicollis	0.0352975	Widespread
Tangara_nigrocincta	0.03574115	Widespread
Tangara_cyanoptera	0.03294677	Widespread
Tangara_heinei	0.00230625000000001	Widespread
Tangara_argyrofenges	0.00115573999999999	Endemic
Tangara_vitriolina	0.0137423	Widespread
Tangara_ruficervix	0.08876813	Widespread
Tangara_vassorii	0.05638682	Widespread

Tangara_nigroviridis	0.04617351	Widespread
Tangara_gyrola	0.02366559	Widespread
Tangara_lavinia	0.05977709	Widespread
Tangara_chilensis	0.03786345	Widespread
Tangara_callophrys	0.02210466	Widespread
Tangara_velia	0.01962161	Widespread
Tangara_mexicana	0.03124464	Widespread
Tangara_inornata	0.04057205	Widespread
Tangara_chrysotis	0.06735951	Widespread
Tangara_xanthocephala	0.0555636	Widespread
Tangara_johannae	0.06426462	Widespread
Tangara_parzudakii	0.07263707	Widespread
Tangara_schrankii	0.04216379	Widespread
Tangara_arthus	0.05753318	Widespread
Tangara_florida	0.04190527	Widespread
Tangara_icterocephala	0.03473608	Widespread
Tangara_cyanotis	0.09210779	Widespread
Tangara_labradorides	0.05248864	Widespread
Tangara_rufigenis	0.08469248	Endemic
Tinamus_major	0.21520348	Widespread
Crypturellus_soui	0.02902302	Widespread
Tinamus_guttatus	0.00212324	Endemic
Trogon_rufus	0.01849652	Widespread
Trogon_collaris	0.01570367	Widespread
Trogon_personatus	0.02120734	Widespread
Trogon_viridis	0.01572243	Widespread
Trogon_violaceus	0.00653333	Widespread
Trogon_curucui	0.00674706	Widespread
Trogon_comptus	0.00939376	Widespread
Trogon_melanurus	0.00510195	Widespread
Trogon_massena	0.00947864	Widespread
Pharomachrus_mocinno	0.01475261	Widespread
Pharomachrus_fulgidus	0.00019757	Widespread
Pharomachrus_antisianus	7.6e-07	Widespread
Pharomachrus_pavoninus	0.00861815	Widespread
Pharomachrus_auriceps	0.00983585	Widespread
Tremarctos_ornatus	0.00378777	Widespread
Mesophylla_macconnelli	0.15581481	Widespread
Vampyressa_melissa	0.12833033	Widespread
Vampyressa_thyone	0.03582936	Widespread
Vampyressa_pusilla	0.07888348	Widespread
Crotalus_basiliscus	0.21951587	Endemic
Crotalus_durissus	0.03132018	Widespread
Lachesis_acrochorda	0.02705623	Widespread

Lachesis_muta	0.01896282	Widespread
Porthidium_nasutum	0.02584055	Widespread
Porthidium_lansbergii	0.02637622	Widespread
Bothrops_pulchra	0.05703412	Widespread
Bothrops_bilineata	0.03011639	Widespread
Bothrops_taeniata	0.03025175	Widespread
Bothrops_punctatus	0.03968678	Widespread
Bothrops_osbornei	0.03446312	Endemic
Bothrops_asper	0.02782816	Widespread
Bothrops_atrox	0.01526565	Widespread
Bothrocophias_microphthalmus	0.02606715	Endemic
Xylocopa_frontalis	0.06711566	Widespread
Xylocopa_fimbriata	0.02149422	Widespread
Zamia_chigua	0.00837147999999999	Widespread
Zamia_gentryi	0.00389983999999999	Endemic
Zamia_restrepoi	0.00470823999999997	Endemic
Zamia_manicata	0.000701469999999982	Widespread
Zamia_poeppigiana	1.40999999997948e-06	Widespread
Zamia_lindenii	1.40999999997948e-06	Widespread
Zamia_encephalartoides	0.00392743000000001	Endemic
Zamia_lecointei	0.00980656000000002	Endemic
Zamia_muricata	0.00817382999999999	Widespread
Zamia_obliqua	0.00977401	Widespread
Zamia_pseudoparasitica	0.01640417	Endemic
