# Supplementary material for: Phylogenetic diversity and North Andean block conservation
Source: PeerJ. 2023 Dec 6;11:e16565. doi: 10.7717/peerj.16565 (PMC10710123; doi:10.7717/peerj.16565)
Supplement: Supplemental Information 2 — The groups utilized in our analyses, the genes employed for phylogenetic reconstruction, and the Digital Object Identifier (DOI) sourced from GBIF for reference. [file peerj-11-16565-s002.pdf]

Group	Number of species		Taxonomic Rank	Genes	GBIF
	Distribution	Terminals			
Acalypha	10	34	Genus	TRNL-F, RBCL, MATK, ITS-18S, PSBA	https://doi.org/10.15468/dl.ehuoxz https://doi.org/10.15468/dl.fbrdmp
Anolis	36	180	Genus	CYT-B, 16S, ND2, COI, RAG1, ITS1-8S	https://doi.org/10.15468/dl.7ftxu8
Aotidae	4	8	Family	COII, COI, CYT-B, SRY, RAG1, POLA1, PNOC, PLCB-4, BRCA-2, ADORA-3; BDNF	https://doi.org/10.15468/dl.dlflps
Aratinga	12	49	Genus	RAG-1, CYT-B, NAD-2, 16S, 12S	https://doi.org/10.15468/dl.akpopk
Ariteus	3	9	Genus	CYT-B, RAG-2, PLCB-4, ATP-7, BDNF, THY, ITS-18S	https://doi.org/10.15468/dl.dlflps
Artibeina	16	24	Subtribe	CYT-B, RAG-2, PLCB-4, ATP-7, BDNF, COI, THY, ITS-18S	https://doi.org/10.15468/dl.dlflps
Atelidae	6	20	Family	COI, COII, CYT-B, SRY, RAG-2, POLA-1, PNOC, PLCB-4, BRCA-2, ADORA-3, BDNF	https://doi.org/10.15468/dl.dlflps
Atractus	9	18	Genus	16S, 12S, C-MOS, NADH-4, CYT-B	https://doi.org/10.15468/dl.7ftxu8
Bactris	12	35	Genus	ITS-18S, TRNL-F, MATK, RPS-16, RPB-2, TRNQ, RBC-L	https://doi.org/10.15468/dl.ehuoxz https://doi.org/10.15468/dl.fbrdmp
Bazzania	6	25	Genus	RBC-L, PSB-A, 26S, NADH-1	https://doi.org/10.15468/dl.pw758k
Bomarea	25	55	Genus	MATK, PSB-A, RBC-L, ITS-18S, RPOB, NADH-F. MATR	https://doi.org/10.15468/dl.ehuoxz https://doi.org/10.15468/dl.fbrdmp
Bombus	8	52	Genus	EF-1, PEPCK, 16S	https://doi.org/10.15468/dl.jl4awt
Caesalpinia	6	47	Genus	RPS-16, MARK, RBC-L, ITS-18S, ITS-5.8 S	https://doi.org/10.15468/dl.ehuoxz
Calamagrostis	3	32	Genus	TRNK-PSBA, RBC-L, ITS-18S, TRNL-F	https://doi.org/10.15468/dl.ehuoxz https://doi.org/10.15468/dl.fbrdmp
Calceolaria	26	100	Genus	ITS-18S, MATK, RBC-L	https://doi.org/10.15468/dl.ehuoxz https://doi.org/10.15468/dl.fbrdmp
Caligo	7	11	Genus	COI, CAD, RPS5, GAPDH, EF-1A	https://doi.org/10.15468/dl.jl4awt
Canidae	5	36	Family	COI, COII, CYT-B, APOB, GHR, CHRNA-1, PNOC, RAG-1	https://doi.org/10.15468/dl.dlflps
Canthon	5	14	Genus	28S, 18S, 16S, COI	https://doi.org/10.15468/dl.jl4awt
Capitonidae	8	13	Family	CYT-B, NADH2, COI, FB-7	https://doi.org/10.15468/dl.akpopk
Catasticta	2	7	Genus	COI, EF-1A, WING	https://doi.org/10.15468/dl.ehuoxz https://doi.org/10.15468/dl.fbrdmp
Cathartidae	5	8	Family	COI, RAG-1, 12S, CYT-B, PER2	https://doi.org/10.15468/dl.akpopk
Cebidae	4	41	Family	COII, COI , CYT-B, SRY, RAG-2, RAG-1, POLA-1, PNOC, PLCB-4, BRCA-2, ADORA3, BDNF	https://doi.org/10.15468/dl.dlflps
Ceroxylon	9	13	Genus	ATPH-I, ATPH-F, NADH-F, PRK	https://doi.org/10.15468/dl.ehuoxz https://doi.org/10.15468/dl.fbrdmp
Chiroderma	3	6	Genus	CYT-B, ATP-7A, RAG-2, BDNF, COI	https://doi.org/10.15468/dl.dlflps
Chironius	6	18	Genus	CYT-B, 12S, 16S, C-MOS	https://doi.org/10.15468/dl.7ftxu8
Chlorochrysa	7	13	Genus	CYT-B, NADH-2, COI, FGB-5, RAG-1	https://doi.org/10.15468/dl.akpopk
Christensonella	2	15	Genus	ITS-18S, TRNL-F, MATK, RCB-L	https://doi.org/10.15468/dl.ehuoxz https://doi.org/10.15468/dl.fbrdmp
Chusquea	29	79	Genus	NADH-F, ITS-18S, TRN-L, NADH-A, RPL-16, TRNQ	https://doi.org/10.15468/dl.ehuoxz https://doi.org/10.15468/dl.fbrdmp
Ciconidae	2	4	Family	CYT-B, COI, 12S, FG-I7	https://doi.org/10.15468/dl.akpopk
Columnea	58	85	Genus	PSB-A, ITS-18S, TRNQ, RPS16, MATK	https://doi.org/10.15468/dl.ehuoxz https://doi.org/10.15468/dl.fbrdmp
Cyathea	24	61	Genus	MATK, RBC-L, ATP-B, TRN-L	https://doi.org/10.15468/dl.e3yepj
Deltochilum	3	7	Genus	COI, 16S, 28S	https://doi.org/10.15468/dl.jl4awt
Dendrobatidae	33	104	Family	CYT-B, COI, RHO, RAG-1, 28S, BDNF, H-3	https://doi.org/10.15468/dl.wwuvew
Dendropicinni	12	56	Tribe	BF7, NADH-2, RAG-1, CYT-B, COI, MYO-I2, 12S	https://doi.org/10.15468/dl.akpopk
Desmodontinae	3	4	Subfamily	CYT-B, RAG-2, PLCB-4, ATP-7, BDNF, COI	https://doi.org/10.15468/dl.dlflps
Didelphimorphia	29	80	Order	CYT-B, COI, NADH-2, VWF, IRBP, RAG-1, BRCA-1	https://doi.org/10.15468/dl.dlflps
Diplazium	17	104	Genus	ATP-A, ATP-B, RBC-L, MATK,TRNL-F	https://doi.org/10.15468/dl.zcyfwj
Dipsas	6	17	Genus	12S, 16S, C-MOS, NADH-4, CYT-B, BDNF	https://doi.org/10.15468/dl.7ftxu8
Espeletia	22	46	Genus	ITS-18S, ACC-D, MATK, ATP-B, RBC-L	https://doi.org/10.15468/dl.ehuoxz https://doi.org/10.15468/dl.fbrdmp
Euglossini	38	85	Tribe	CYT-B, COI, EF-1A, ARG-K, POL-2	https://doi.org/10.15468/dl.jl4awt
Falco	6	34	Genus	RAG-1, CYT-B, PEPCK, COI	https://doi.org/10.15468/dl.akpopk
Felidae	7	35	Family	ATP-A, COI, CYT-B, SRY, RAG-2, RAG-1, PNOC, PLCB-4, BRCA-1, ADORA3, BDNF, 12S, 16S, NADH-2, NADH-5	https://doi.org/10.15468/dl.dlflps
Fregata	1	6	Genus	CYT-B, COI, NADH-2, FG-I7, 12S	https://doi.org/10.15468/dl.akpopk

Geonoma	31	47	Genus	RPS16, RPB2, PRK, CISP-4, RBC-L	https://doi.org/10.15468/dl.ehuoxz https://doi.org/10.15468/dl.fbrdmp
Glossophaginae	19	30	Subfamily	CYT-B, RAG-2, PLCB4, ATP-7, BDNF, COI	https://doi.org/10.15468/dl.dlflps
Ephedra	4	56	Genus	MATK, ATP-B, RBC-L, ITS-18S, TRNL-F, COI	https://doi.org/10.15468/dl.bu68de
Gonatodes	8	18	Genus	12S, 16S, C-MOS, RAG2	https://doi.org/10.15468/dl.7ftxu8
Habenaria	11	42	Genus	ITS-18S, MATK, TRN-K, RBC-L	https://doi.org/10.15468/dl.ehuoxz https://doi.org/10.15468/dl.fbrdmp
Heliconius	22	43	Genus	COI-COII, EF-1A, WINGLESS, IDH, GAPDH, CAD	https://doi.org/10.15468/dl.jl4awt
Hylidae	72	185	Family	16S, RAG-1, CYT-B, POMC, 12S, COI	https://doi.org/10.15468/dl.zuuty7
Kinosternon	3	18	Genus	COI, CYT-B, C-MOS, 16S, RAG-1	https://doi.org/10.15468/dl.7ftxu8
Leptodeira	8	18	Genus	NADH-4, C-MOS,CYT-B, 16S, 12S	https://doi.org/10.15468/dl.7ftxu8
Lupinus	16	72	Genus	ITS-18S, TRNL-F, GLYCEROL3, MATK, RBC-L	https://doi.org/10.15468/dl.ehuoxz https://doi.org/10.15468/dl.fbrdmp
Lutrinae	2	14	Subfamily	CYT-B, RAG-1, RAG-2, BRCA-1, BDNF, RHO	https://doi.org/10.15468/dl.dlflps
Lycopodiaceae	25	86	Family	RBC-L, PSB-A, TRNL	https://doi.org/10.15468/dl.zn79t3
Macleania	8	9	Genus	MATK, ITS-18S, NADH-F	https://doi.org/10.15468/dl.ehuoxz https://doi.org/10.15468/dl.fbrdmp
Trochilidae	11	21	Family	ODC, CYT-B, NADH-2, COI, BFIB	https://doi.org/10.15468/dl.akpopk
Mastigodryas	4	7	Genus	CYT-B, 12S, 16S, C-MOS	https://doi.org/10.15468/dl.7ftxu8
Maxillaria	31	52	Genus	ITS-18S, MATK, ATP-B, RPO-C1	https://doi.org/10.15468/dl.ehuoxz https://doi.org/10.15468/dl.fbrdmp
Megascops	8	22	Genus	COI, RAG-1, CYT-B, MUSK, FGB, NADH2	https://doi.org/10.15468/dl.akpopk
Melocactus	1	5	Genus	MATK, PHOCAR, RPL16,TRNF-L	https://doi.org/10.15468/dl.ehuoxz https://doi.org/10.15468/dl.fbrdmp
Mephitidae	1	10	Family	COI, CYT-B, FES, APOB, GHR, RHO1, RHO2, NADH2, CHRNA-1, ATP-7, APP, ADORA3, PNOC, RAG-2	https://doi.org/10.15468/dl.dlflps
Metallura	5	15	Genus	ODC, CYT-B, NADH-2, COI, FGB	https://doi.org/10.15468/dl.akpopk
Micrathena	3	22	Genus	COI, 16S, ITS-18S	https://doi.org/10.15468/dl.kk65e9
Micrurus	9	27	Genus	ITS-18S, CYTB, C-MOS	https://doi.org/10.15468/dl.7ftxu8
Momotidae	5	7	Family	CYT-B, NADH-2, COI, FB-I7	https://doi.org/10.15468/dl.akpopk
Mormoopidae	5	9	Family	COI, CYT-B, APOB, NADH-2, RAG-2, PLCB4, BDNF	https://doi.org/10.15468/dl.dlflps
Morpho	10	31	Genus	COI, CYT-B, CAD, RPS-5, GAPDH, MDH, EF-1A	https://doi.org/10.15468/dl.jl4awt
Mustelinae	3	36	Subfamily	CYT-B, RAG-1, RAG-2, BRCA-1, BDNF, RHO	https://doi.org/10.15468/dl.dlflps
Napeogenes	9	22	Genus	EF-1A, TEKTIN, COI-COII	https://doi.org/10.15468/dl.jl4awt
Natalidae	3	8	Family	COI, RAG-2, CYT-B	https://doi.org/10.15468/dl.dlflps
Noctilionoidea	5	7	Superfamily	COI, PLCB-4, RAG-2, BDNF, PNOC	https://doi.org/10.15468/dl.dlflps
Opuntia	9	100	Genus	RBC-L, MATK, ATP-B	https://doi.org/10.15468/dl.ehuoxz https://doi.org/10.15468/dl.fbrdmp
Ortalis	4	10	Genus	CYT-B, 12S, FB-I7, NADH-2, RAG-1, RAG-2, COI	https://doi.org/10.15468/dl.akpopk
Pereskia	1	10	Genus	RBC-L, TRNK, RPL16, COIII, PSB-A	https://doi.org/10.15468/dl.ehuoxz https://doi.org/10.15468/dl.fbrdmp
Phyllostominae	21	43	Subfamily	CYT-B, RAG-2, PLCB-4, ATP-7, BDNF, COI, THY, ITS-18S	https://doi.org/10.15468/dl.dlflps
Piper	51	103	Genus	ITS-18S, PSB-A, NADH-F, TRNL-F, TRN-H, MATK, RBC-L	https://doi.org/10.15468/dl.ehuoxz https://doi.org/10.15468/dl.fbrdmp
Pitheciidae	3	27	Family	COII, COI, CYT-B, SRY, RAG-2, POLA1, PNOC, PLCB-4, BRCA-2, ADORA3, BDNF	https://doi.org/10.15468/dl.dlflps
Platyrrhinus	13	17	Genus	CYT-B, RAG-2, PLCB-4, ATP-7, BDNF, COI, THY, ITS-18S, NADH-2	https://doi.org/10.15468/dl.dlflps
Podiceps	3	10	Genus	NADH-2, COI, RAG-1	https://doi.org/10.15468/dl.akpopk
Pristimantis	79	123	Genus	COI, 12S, 16S, RAG-1, TYR	https://doi.org/10.15468/dl.6qs5tr
Procyonidae	9	13	Family	CYT-B, COI, RAG-2, BDNF, RAG-1, ATP-7, ADORA3, NADH-5, NADH-2, 12S	https://doi.org/10.15468/dl.dlflps
Ramphastidae	20	35	Family	CYT-B, NADH-2, COI, FG-I7	https://doi.org/10.15468/dl.akpopk
Sphagnum	32	161	Genus	TRNG, RBC-L, MATK, ITS-18S, RPL16, TRNL, ILY	https://doi.org/10.15468/dl.bbbhtc
Sturnira	10	13	Genus	CYT-B, RAG-2, RAG-1, NADH-2, COI	https://doi.org/10.15468/dl.dlflps
Tangara	32	51	Genus	CYT-B, NADH-2, COI, FG-I5 RAG-1	https://doi.org/10.15468/dl.akpopk
Tinaminae	3	6	Subfamily	COI, BDNF, CYT-B	https://doi.org/10.15468/dl.akpopk
Trogonidae	14	17	Family	BF-7, NADH-2, RAG-1, CYT-B, COI, MYO-I2	https://doi.org/10.15468/dl.akpopk

Ursidae	1	9	Family	ATP-12, COI, CYT-B,SRY, 12S, 16S, NADH-2, ZFY	https://doi.org/10.15468/dl.dlflps
Vampyressa	4	8	Genus	CYT-B, RAG-2, PLCB-4, ATP-7, BDNF, COI, THY, ITS-18, NADH-2	https://doi.org/10.15468/dl.dlflps
Viperidae	13	90	Family	CYT-B, NADH-4, 12S, 16S	https://doi.org/10.15468/dl.7ftxu8
Xylocopa	2	16	Genus	CYT-B, PEPCL, COI, EF-1A, 18S, 28S	https://doi.org/10.15468/dl.jl4awt
Zamia	11	25	Genus	ITS-18S, ATP-B, ATP-F, TRNL, PHYP	https://doi.org/10.15468/dl.88hq9p
