# Supplementary material for: Phylogenetic diversity and North Andean block conservation
Source: PeerJ. 2023 Dec 6;11:e16565. doi: 10.7717/peerj.16565 (PMC10710123; doi:10.7717/peerj.16565)
Supplement: Supplemental Information 3 — The mean probability of success was calculated based on 10 iterations for three different numbers of randomly selected phylogenies. [file peerj-11-16565-s003.pdf]

No of Phylogenies	Mean probability of classification success (p/N)				
	Q1	Q2	Q3	Q4	Q5
23	0.76	0.42	0.37	0.37	0.41
46	0.88	0.63	0.63	0.63	0.69
69	0.93	0.80	0.81	0.89	0.97
