# Supplementary figures and images for: Phylogenetic diversity and North Andean block conservation
Source: PeerJ. 2023 Dec 6;11:e16565. doi: 10.7717/peerj.16565 (PMC10710123; doi:10.7717/peerj.16565)

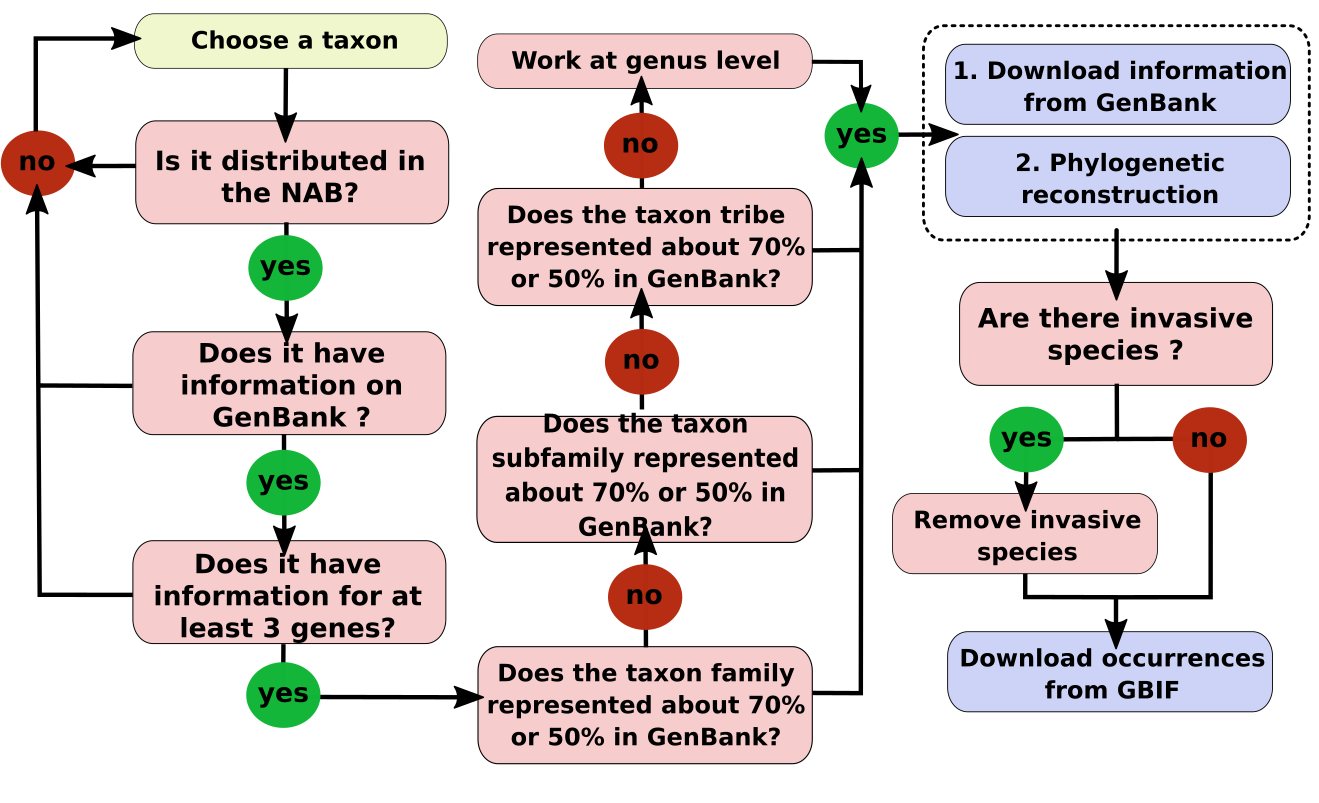

Supplement: Supplemental Information 4 — The rules used to select the taxa for this study. [file peerj-11-16565-s004.png]

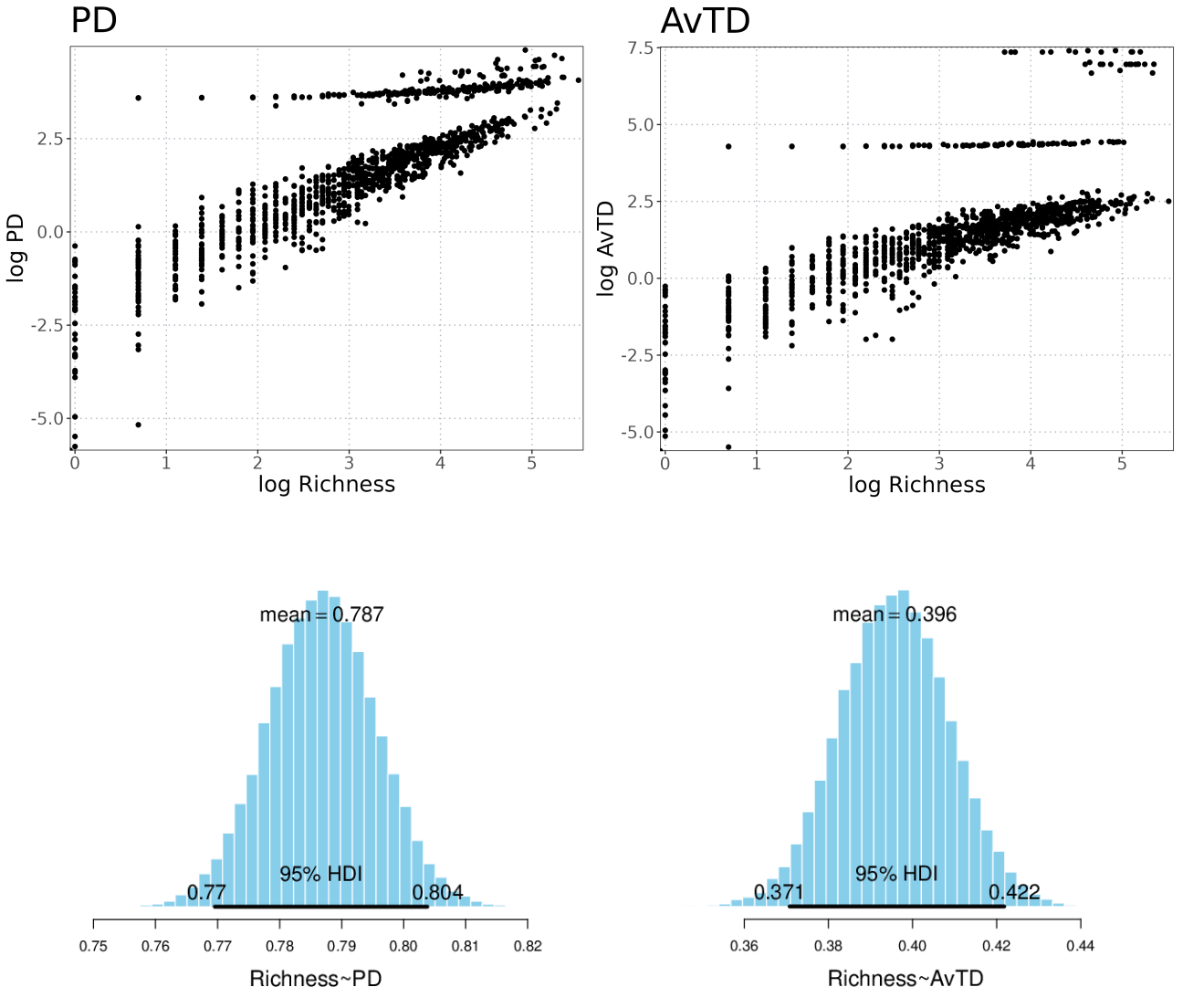

Supplement: Supplemental Information 5 — The results of the Bayesian Linear Regression between Richness and both indices, PD and AVTD, are presented. The upper section displays a graphical representation of both variables, while the lower section illustrates the posterior distribution of the regression slope, depicting the mean slope value. [file peerj-11-16565-s005.png]

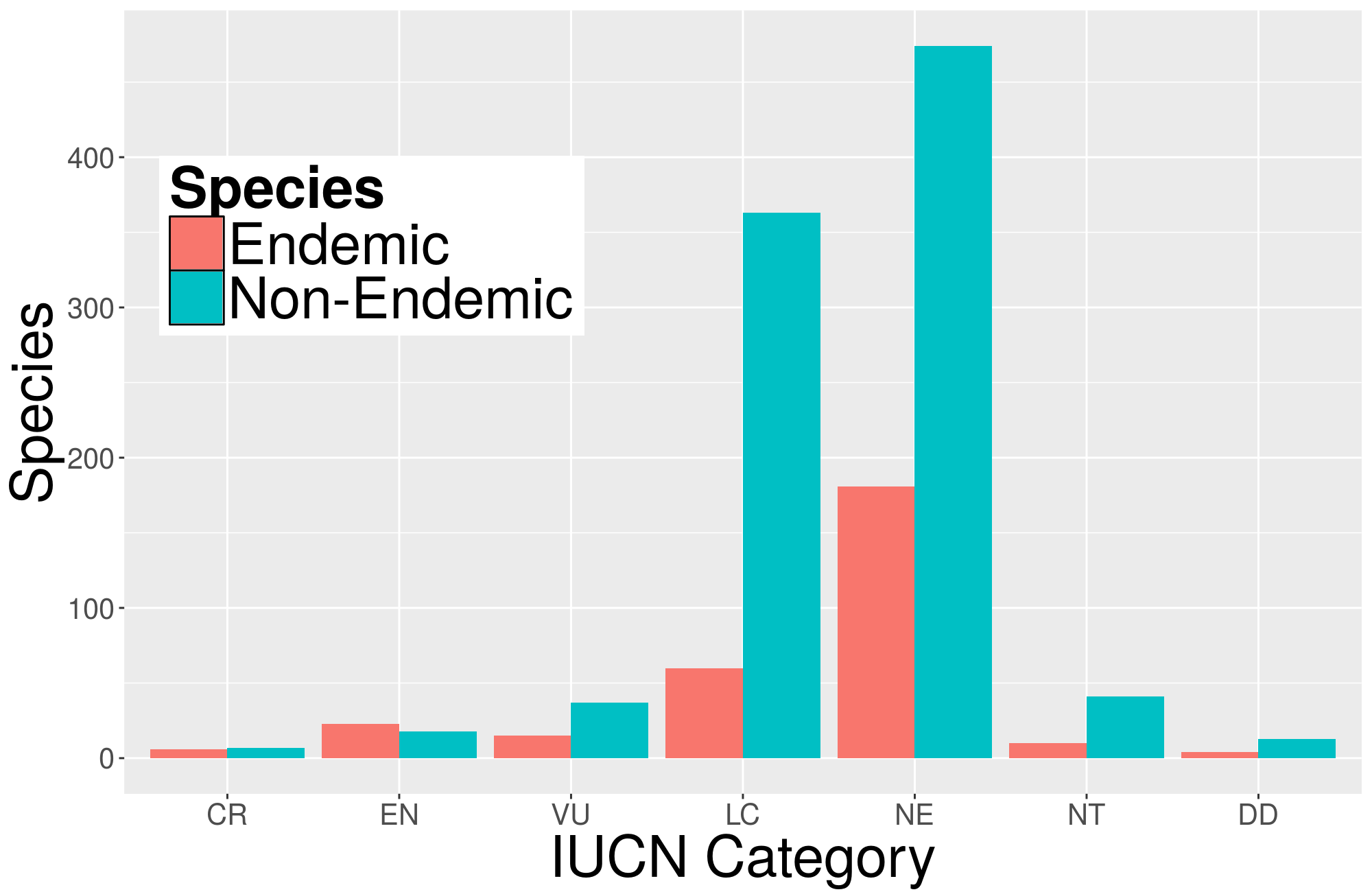

Supplement: Supplemental Information 6 — The number of species in each IUCN category. Most of the species are catalogue as Least Concern (LC) and Not Evaluated (NE). [file peerj-11-16565-s006.png]
